# Design of a Mobile Brain Computer Interface-Based Smart Multimedia Controller

**DOI:** 10.3390/s150305518

**Published:** 2015-03-06

**Authors:** Kevin C. Tseng, Bor-Shing Lin, Alice May-Kuen Wong, Bor-Shyh Lin

**Affiliations:** 1 Department of Industrial Design, Chang-Gung University, Taoyuan 333, Taiwan; E-Mail: ktseng@pddlab.org; 2 Healthy Aging Research Centre, Chang Gung University, Taoyuan 333, Taiwan; 3 Department of Computer Science and Information Engineering, National Taipei University, New Taipei 237, Taiwan; E-Mail: bslin@mail.ntpu.edu.tw; 4 Department of Physical Medicine and Rehabilitation, Chang Gung Medical Foundation, Taoyuan 333, Taiwan; E-Mail: walice@adm.cgmh.org.tw; 5 Institute of Imaging and Biomedical Photonics, National Chiao Tung University, Tainan 711, Taiwan; 6 Department of Medical Research, Chi-Mei Medical Center, Tainan 710, Taiwan

**Keywords:** brain computer interface, multimedia controller, electroencephalograph, music biofeedback

## Abstract

Music is a way of expressing our feelings and emotions. Suitable music can positively affect people. However, current multimedia control methods, such as manual selection or automatic random mechanisms, which are now applied broadly in MP3 and CD players, cannot adaptively select suitable music according to the user’s physiological state. In this study, a brain computer interface-based smart multimedia controller was proposed to select music in different situations according to the user’s physiological state. Here, a commercial mobile tablet was used as the multimedia platform, and a wireless multi-channel electroencephalograph (EEG) acquisition module was designed for real-time EEG monitoring. A smart multimedia control program built in the multimedia platform was developed to analyze the user’s EEG feature and select music according his/her state. The relationship between the user’s state and music sorted by listener’s preference was also examined in this study. The experimental results show that real-time music biofeedback according a user’s EEG feature may positively improve the user’s attention state.

## 1. Introduction

Music is a significant approach to enhancing human physiology and psychology and applied in many areas such as disease treatment and recovery, physical rehabilitation, and enrichment of cognitive function. For example, music positively affects people with dementia, heart disease, or stroke [1,2], increases the effectiveness of pain alleviation and relaxing during rehabilitation [3], and enables people to improve mood state and mental processing [4]. Recently, many studies have explored the relationship between music listening and task performance. For instance, some studies indicated that music positively enhances work performance [5,6,7].

However, current multimedia control methods, such as manual selection or automatic random mechanisms, which are now applied broadly in MP3 and CD players, cannot adaptively select music according to the user’s physiological state. In order to achieve this aim, a novel brain computer interface (BCI)-based smart multimedia controller is proposed in this study. Unlike manual selection or automatic random mechanism, our BCI-based smart multimedia controller selects music types according to the user’s cognitive state. Brain computer interface provides a pragmatic and non-invasive way for communication between the human brain and external devices [8,9,10,11,12,13,14]. Several BCI-based controllers have been proposed in previous studies; however, only a few studies have tried to explore it on music control [15]. However, the above BCI-based multimedia controllers require the user’s active mental command to control multimedia, but cannot select music automatically and adaptively according to the user’s current state. Moreover, most of BCI systems require bulky and expensive electroencephalograph (EEG) machines and personal computers to process EEG in real time. This also limits the feasibility of these BCI-based multimedia controllers for daily applications.

With the rapidly increasing popularity of mobile tablet, using mobile tablets as assistive technology devices of music listening becomes feasible and convenient. Mobile tablets contain small-volume and light-weight properties. In particular, recently, mobile tablets tend to offer more advanced computing ability, connectivity, and music efficiency. Therefore, in the proposed BCI-based multimedia controller, a commercial mobile tablet was used as the development platform. A smart multimedia control program built in the mobile tablet was also developed to continuously monitor the user’s EEG feature. When a music selection nears its end, the smart multimedia control program will select the next music type according to the user’s EEG feature. Moreover, unlike other bulky EEG machines, a wireless multi-channel EEG acquisition module was also designed to provide portability and improve the convenience of use for daily applications.

## 2. System Architecture and Design

**Figure 1:**
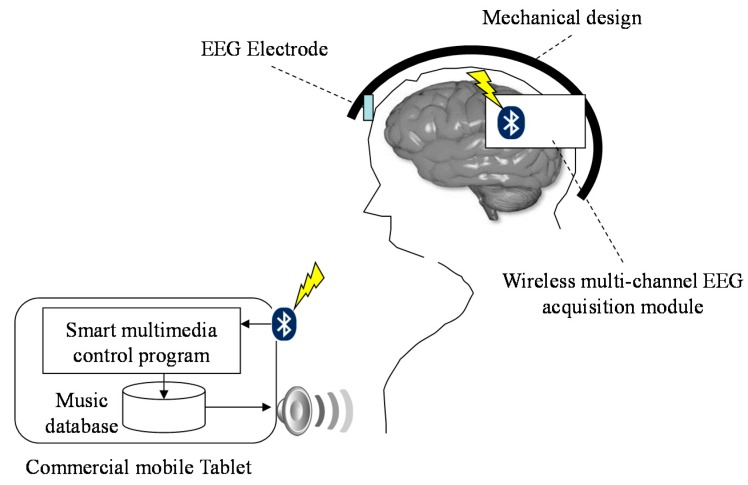
Basic scheme of proposed brain computer interface-based mobile multimedia controller.

The basic scheme of the proposed brain computer interface-based smart multimedia controller is illustrated in Figure 1. The system hardware contains a wireless multi-channel EEG acquisition module and a multimedia platform. Here, the wireless multi-channel EEG acquisition module is designed to acquire multi-channel EEG signals simultaneously and then transmit these EEG signals to the multimedia platform wirelessly. A smart multimedia control program is built in the multimedia platform to receive and analyze the acquired EEG signal and then store the estimated EEG features continuously. Several types of music are stored in different folders on the multimedia platform. When the music section nears its end, the next musical piece will be selected according to the user’s EEG feature.

### 2.1. Wireless Multi-Channel EEG Acquisition Module

**Figure 2:**
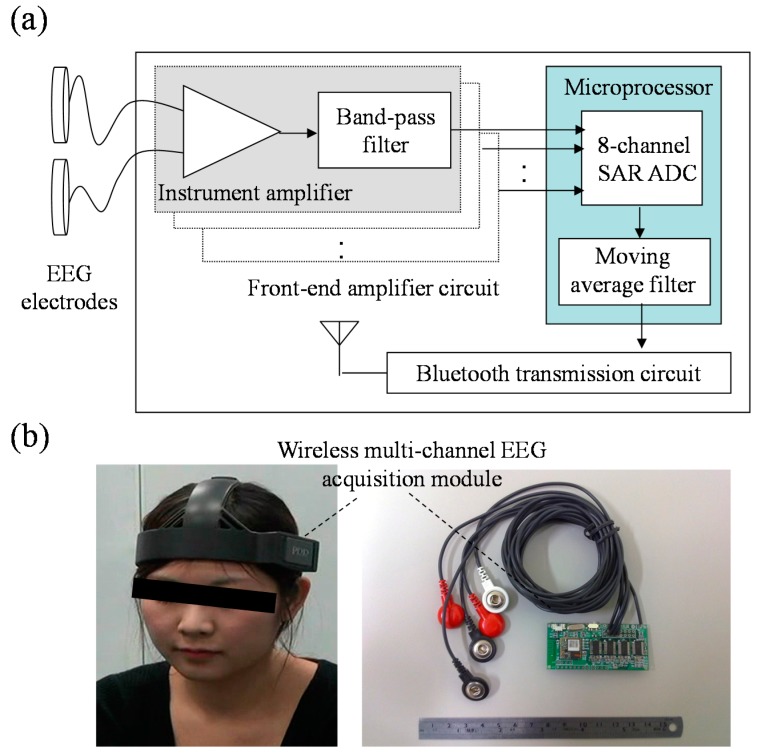
(**a**) Block diagram and (**b**) photograph of wireless multi-channel EEG acquisition module.

The block diagram of the wireless multi-channel EEG acquisition module, as shown in Figure 2a, consists of three main parts: The front-end amplifier circuit, the microprocessor, and the Bluetooth transmission circuit. Here, the front-end amplifier circuit is designed to amplifier and filter EEG signals acquired from EEG electrodes, and the microprocessor is used to digitize the EEG signal, perform a low-pass filter, control the peripheral circuits, and send EEG signal into the Bluetooth transmission circuit. The front-end amplifier circuit contains an instrument amplifier and a band-pass filter. The gain of the front-end amplifier circuit is set to 5000 times with a frequency band of 0.1 Hz~150 Hz. In this study, an ultra-low power 16-bit RISC mixed-signal microprocessor (MPS430, Texas Instruments, Dallas, TX, USA) is used as the microprocessor. It can provide an 8-channel 12-bit successive approximation register analog-to-digital converter (SAR ADC) used to digitize acquired EEG signal. Here, the sampling rate of the SAR ADC is set to 512 Hz. Before transmitting the EEG signal to the Bluetooth transmission circuit, the microprocessor will pass the EEG signal through a moving average with a 56 Hz cutoff frequency to reduce the power-line interference. The Bluetooth transmission circuit contains a printed circuit board (PCB) antenna and a Bluetooth module, which is fully compliant with the Bluetooth v2.0+ EDR specification. The microprocessor communicates with the Bluetooth module via a universal asynchronous receiver/transmitter (UART). The wireless multi-channel EEG acquisition module is designed to operate at 35 mA with a 3.7 V DC power supply, and can be operated continuously for over 30 h with a commercial 1100 mAh Li-ion battery. The size of the wireless multi-channel EEG acquisition module is about 62 mm × 28 mm × 5 mm. Figure 2b shows the photograph of the wireless multi-channel EEG acquisition module.

### 2.2. Multimedia Platform

In this study, a commercial mobile tablet is used as the multimedia platform, and the operation system (OS) of the multimedia platform is Windows 8.1. The operation system of Windows 8.1 is known to allow the multi-tasking, allowing users to operate more than one application at one point of time. Besides the basic functions of receiving, displaying and storing the raw EEG data, the smart multimedia control program built in the mobile tablet will also provide the functions of monitoring the user’s EEG feature and selecting next music type according the user’s EEG feature.

## 3. System Software Design

### 3.1. Implement of Smart Multimedia Control Program

**Figure 3:**
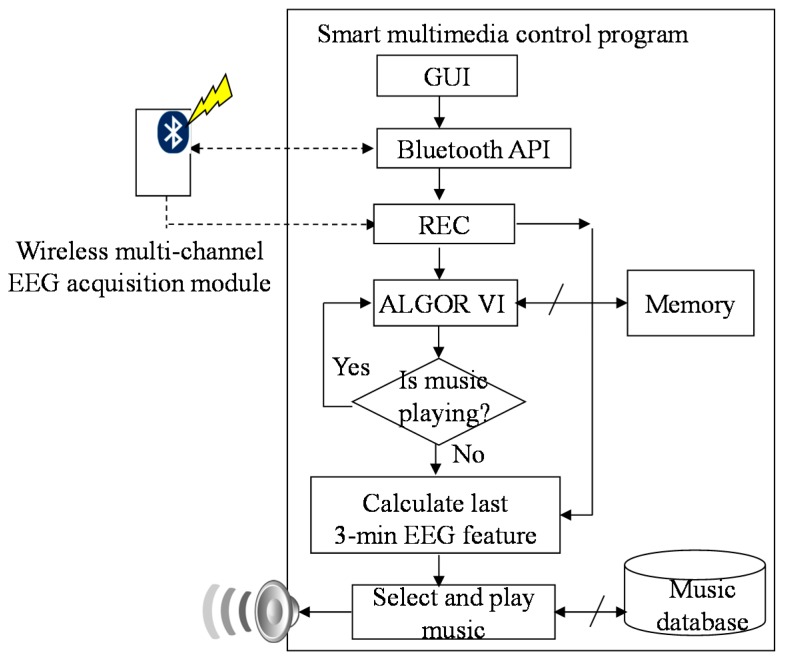
Operation procedure of smart multimedia control program.

The smart multimedia control program is developed by Microsoft C#. The operation procedure of the smart multimedia control program is shown in Figure 3. In the beginning, the smart multimedia control program will build the graphical user interface (GUI). Next, the smart multimedia control program will call the thread of Bluetooth API to search the wireless multi-channel EEG acquisition module and create a SPP stream to connect with the module, and then the REC thread will receive the raw EEG signal and store them into the memory and the solid-state drive (SSD) of the tablet. Next, the ALGOR thread will calculate EEG feature from EEG signal recorded in the locations Fz, Fp1 and Fp2 of the EEG 10-20 electrode system. The smart multimedia control program will check the playing state of the music. If the musical piece is nearly over, the smart multimedia control program will calculate the average of the last three-minute EEG features and decide the next music type according to the averaged EEG feature.

### 3.2. EEG Feature Extraction Algorithm

**Figure 4:**
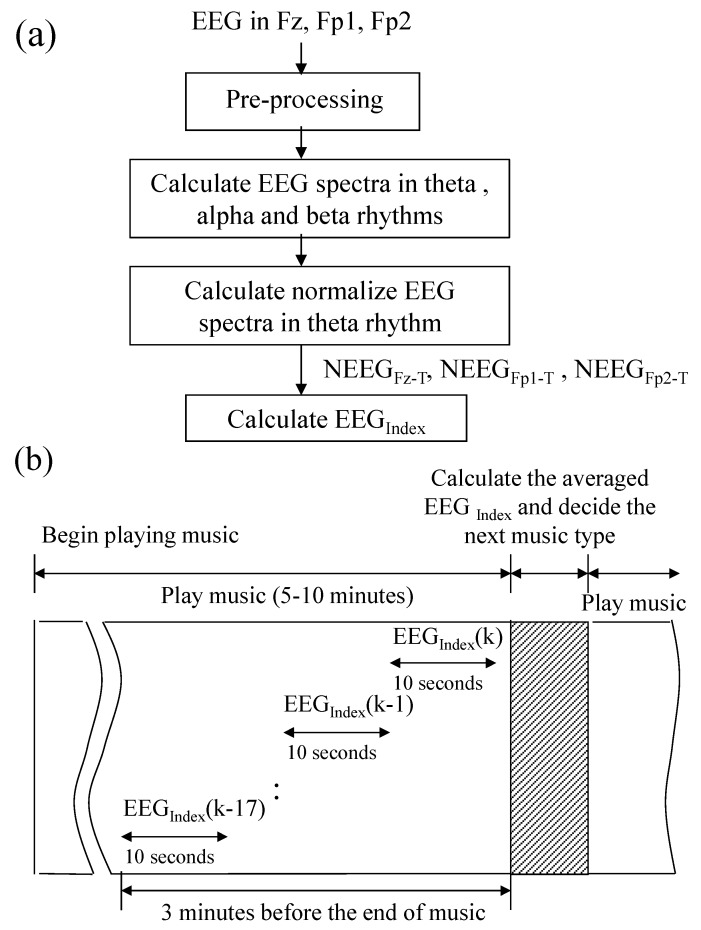
(**a**) Flowchart of EEG feature extraction algorithm, and (**b**) time-series diagram of multimedia control.

Changes in the sustained necessary attention can be reflected in the EEG spectra as theta rhythm (4~7 Hz) [16,17]. Iramina *et al.* have investigated the relationship between the measured EEG signals and subject’s level of attention state, and they found that frontal theta increases when participants were studying but decreases when resting. Meanwhile, frontal alpha acts oppositely [16]. Moreover, Ishii *et al.* indicated that EEG spectra in the theta rhythm, obtained from the frontal midline, can provide discriminating power and has a high correlation with sustained necessary attention [17]. Based on the abovementioned phenomenon, EEG feature extraction algorithm was designed (its flowchart is shown in Figure 4a). First, EEG signals recorded from the locations Fz, Fp1, and Fp2 will be preprocessed. Here, EEG signal is down-sampled to 64 Hz to reduce the computation load, and a 512-point fast Fourier transform (FFT) with a 448-point overlap is used to obtain EEG spectra. Next, EEG spectra in theta, alpha (8~12 Hz), and beta (13~30 Hz) rhythms are calculated. In order to reduce the influence of measuring difference from session to session or from subject to subject, the normalized EEG spectra in theta rhythm is calculated. Let $EEG_{Fz-T}(t)$, $EEG_{Fz-A}(t)$, and $EEG_{Fz-B}(t)$ be EEG spectra, obtained from Fz at iteration t, in theta, alpha, and beta rhythms, respectively. The normalized EEG spectra in theta rhythm can be calculated with the following equation:

(1) $$NEEG_{Fz-T}(t)=\frac{EEG_{Fz-T}(t)\times 100\%}{EEG_{Fz-T}(t)+EEG_{Fz-A}(t)+EEG_{Fz-B}(t)}$$

Next, a linear combination $EEG_{Index}(t)$ of normalized EEG spectra in theta rhythm at iteration t can be calculated by:

(2) $$EEG_{Index}(t)=NEEG_{Fz-T}(t)+NEEG_{Fp1-T}(t)+NEEG_{Fp2-T}(t)$$

where $NEEG_{Fp1-T}(t)$ and $NEEG_{Fp2-T}(t)$ denote the normalized EEG spectra in theta rhythm at iteration t, obtained from Fp1 and Fp2 respectively. Here, the reference electrode was placed at the ear. The tendency of the user’s EEG spectra in theta rhythm may remain higher when he/she remains in the state of attention. Otherwise, the tendency of the user’s EEG spectra may decrease, if he/she trends toward the state of inattention. Although the change of the user’s EEG spectra in theta rhythm is not precisely correlated to the change of the user’s state, the change tendency of EEG spectra can still provide a reference index for the user’s state. The time-series diagram of multimedia control is shown in Figure 4b. In order to estimate the tendency of EEG spectra in theta rhythm, the averaged $EEG_{Index}$ for the last three minutes is calculated.

## 4. Experiment Designs

### 4.1. Experiment Design for Effect of Selecting Music Type on Cognitive State

Before music biofeedback, the effect of selecting music type on cognitive state has to be first investigated. Defining a precise music recommendation rule which matches everyone’s requirement is difficult because of differences in cultures, backgrounds, and personalities. Several previous studies attempted to conduct a practical approach to recommend suitable music for the users. Shan *et al.* proposed using the user’s psychology, such as emotions, as the music selection rule because selecting suitable music can enhance the both the psychological and physiological response of the listeners [18]. Levitin and McGill suggested using the specific features of music, such as tempo, rhythm, melody, or the preference, as the music selection rule in different situations [19]. In this study, the preference is used as the music selection factor. Two predefined music selections, such as high focus and piano concerto K488, and a self-selected (SS) musical piece were used in this experiment. Here, high focus is usually composed for brain wave therapy, and K488, composed by Mozart, is the most used concerto in previous studies [20,21].

In this experiment, the preference rate for different music and the self-rating cognitive state level after listening different music were investigated. A total of 28 participants, aged from 18 to 32 years old, attended this experiment. Before the experiment, a staff instructed the participants to choose a favorite piece of music for this test and answer the self-rating cognitive state questionnaire to record the initial cognitive state. The participant has to rate the preference scale for the three music pieces. In the experiment, three types of music piece (high focus, K488, and self-selected music) were tested individually for every participant, and the time duration of these music piece are about 5 min. The participant’s EEG signal was recorded continuously during the entire experimental procedure in a soundproof room that minimized the influence of background noise. When the music piece was over, the participants were instructed to answer the self-rating cognitive state questionnaire again. Here, according to the definition of preference level in continuous digital response interface (CDRI), the preference level for music was rated from 0 to 256. The higher value of preference level denotes more positive responses for the participant. The attention state level was rated from 0 to10 (0: No attention at all, 10: Maximum attention). One-way ANOVA was used to test the significant difference between different music type, and the significance was set at *p* < 0.05.

### 4.2. Experiment Design for Effect of Music Biofeedback

In this experiment, the effect of real-time music biofeedback was examined. Here, a total of 28 participants attended this experiment. The participants were also instructed to rate the preference level for three music types (high focus, K488, and self-selected music). The most preferred music was set as type1 music, the least preferred music was set to type 3, and the neutral music was set as type 2 music. Here, the aim of multimedia control criterion was set to enhance the participant’s attention. Under the assumption of that the higher preference-level music can effectively evoke the user’s attention, the preferred music will be selected when the cognitive status of the participant becomes relatively inattentive. Therefore, the multimedia control criterion was set as follows: (a) select type 1 music as the next piece when the averaged *EEG_Index_* decreases over 5% of the previous averaged *EEG_Index_*; (b) select type 3 music as the next piece when the averaged *EEG_Index_* increases over 5% of the previous averaged *EEG_Index_*; and (c) select type 2 music as the next piece when the change of the averaged *EEG_Index_* is between ±5% of the previous averaged *EEG_Index_*. In this experiment, the initial music was randomly selected. The proposed BCI-based smart multimedia controller was used to monitor the user’s real-time EEG continuously during the entire experiment procedure and to select the next musical piece according to the user’s EEG feature. The time interval between two music pieces is about 5 s. Moreover, after finishing a music piece, the participant will be instructed to answer the self-rating cognitive state questionnaire again to evaluate the performance of music biofeedback.

## 5. Results and Discussions

### 5.1. Effect of Selecting Music Type on Cognitive State

**Figure 5:**
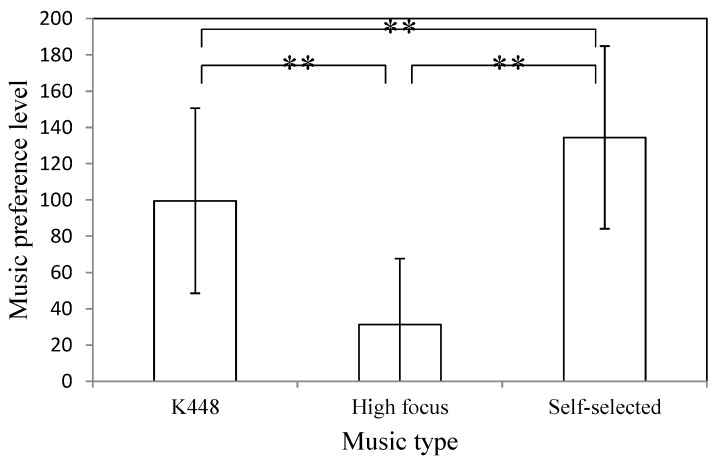
Music preference level corresponding to different types of music. Here, ** denotes significance.

Figure 5 shows the averages and standard deviations of the music preference level corresponding to different music types. The symbol ** denotes significance. The experimental result shows that the difference between the preference levels of any two music types is significant. Therefore, for the participants, the preference levels of high focus, K488, and self-selected music are significantly different.

**Figure 6:**
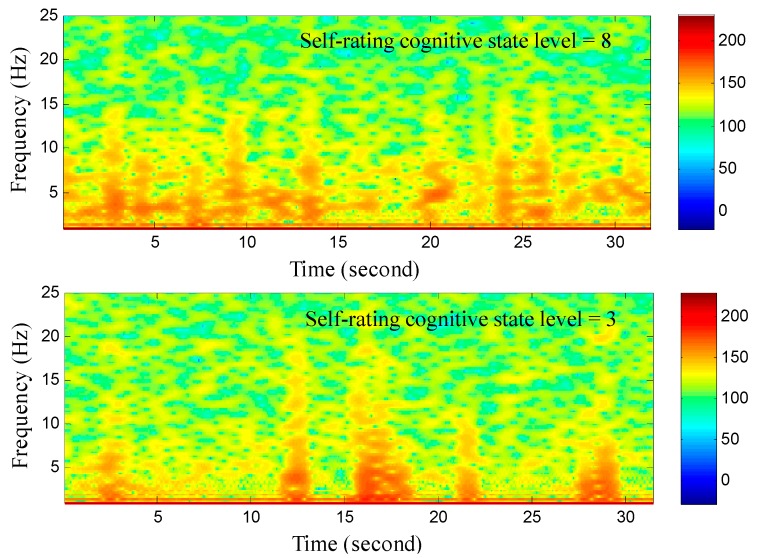
Time-frequency analysis of EEG signal corresponding to different self-rating cognitive state level.

**Figure 7:**
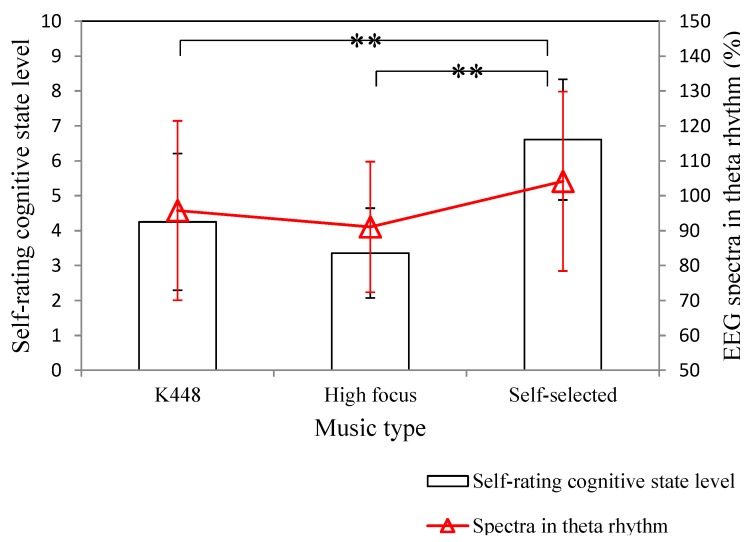
Self-rating cognitive state level and EEG spectra in theta rhythm corresponding to different music types. Here, ** denotes significance.

Next, the effect of listening different music type on the self-rating cognitive state level and EEG spectra in theta rhythm was investigated. Figure 6 shows two experimental results for the time-frequency analysis of EEG signal under different self-rating cognitive state level. The EEG spectra in theta rhythm obviously increases when the self-rating cognitive state level is relatively large. Figure 7 shows the averages and standard deviations of the participants’ cognitive state level and EEG spectra in theta rhythm for different music types. The experimental result shows that the effect of listening self-selected music on evoking attention is most significant. Moreover, the cognitive state level of the participant can also reflected on the EEG spectra in theta rhythm. When the cognitive state level is relatively large, the EEG spectra in theta rhythm exactly increases. The effect of music type on evoking the participant’s attention can also be improved with the increase of the music preference level.

### 5.2. Effect of Music Biofeedback by Using BCI-Based Multimedia Controller

**Figure 8:**
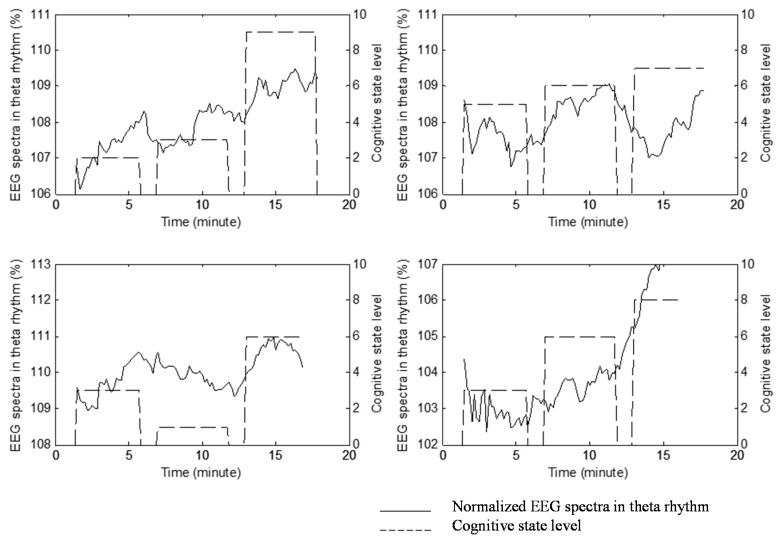
Four randomly selected results for real-time music biofeedback.

**Figure 9:**
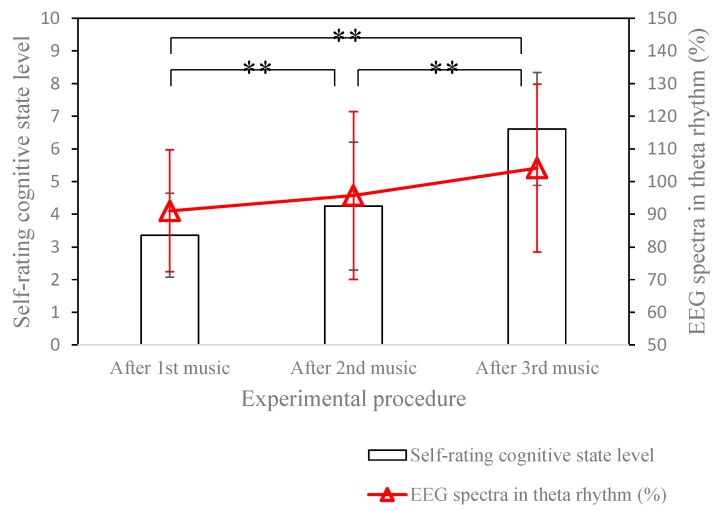
Self-rating cognitive state level and EEG spectra in theta rhythm under music biofeedback. Here, ** denotes significance.

Figure 8 shows four randomly selected results for real-time music biofeedback. As our expectation, most of the participants’ cognitive states trends toward the state of attention, and the tendency of the EEG spectrum in theta rhythm also increased gradually. Experimental results show that about 71.40% of the participants felt their cognitive state significantly trends toward an attentive state, and about 67.86% of the participants’ EEG spectra in theta rhythm trend to increase. Figure 9 shows the averages and standard deviations of self-rating cognitive state level and EEG spectra in theta rhythm under music biofeedback. 

The experimental result shows that, after music biofeedback, the cognitive state level of the participant gradually increases, and the EEG spectra in theta rhythm also trends to increase. Next, the experiment of randomly music selection was also examined, and a total of 20 participants attended this experiment. For randomly music selection, experimental results show that about 45% of the participants’ cognitive state significantly trends toward the state of attention. Therefore, real-time music biofeedback using the proposed system exactly improves the user’s attention state.

### 3.3. Specification Comparison

The specification comparisons between our system and other BCI-based multimedia control systems are listed in Table 1. Shyu *et al.* proposed a FPGA-based SSVEP BCI multimedia control system [15]. Instead of personal computers, the real-time SSVEP BCI algorithm was implemented in a FPGA board. Here, the user can control multimedia via SSVEP generated from four visual stimuli, which were labeled as four multimedia command symbols. However, both of the above BCI-based multimedia control systems require external visual stimulating devices and active mental command to control multimedia. Unlike the above system, the proposed BCI-based multimedia controller controls multimedia automatically and adaptively according to the user’s EEG feature. Moreover, different from the most frequently used bulky EEG machine, the properties of small-volume and wireless communication for the wireless multi-channel EEG acquisition module are more suitable for daily application. Furthermore, a commercial mobile tablet is also used as the multimedia platform. The commercial mobile tablet is small, light, and popular, and this may increase the acceptability for general users.

**Table 1:** Specification comparison between proposed system and other BCI-based multimedia controller.

BCI System	SSVEP BCI Multimedia Control [15]	Proposed BCI-Based Multimedia Controller
EEG Signal	SSVEP	EEG spectra in theta rhythm
Transmission	RF transmission	Bluetooth
Power Supply	Power line	3.7 V Li Battery
Sampling Rate	8 kHz	512 Hz
Gain	1000	5000
Analog Filter	20–22 Hz	0.1–100 Hz
Backend Signal Processing Unit	FPGA board	Mobile tablet
Control Mode	Active BCI [22]	Passive BCI [22]
Multimedia Applications	Music	Music

## 6. Conclusions

In this study, a novel brain computer interface-based smart multimedia controller was proposed to select music according the user’s EEG feature. Unlike other BCI-based multimedia controllers that require the user’s active mental command to control multimedia, the proposed system can control multimedia automatically and adaptively according to the user’s EEG feature. From the experimental results, the effect of the most preferred music on evoking attention is exactly most significant. By using the approach of real-time music biofeedback, the proposed BCI-based smart multimedia controller can positively improve the user’s attention state. Moreover, in this study, the efficiency of selecting music according to the user’s state on evoking attention is superior to that of randomly music selection. The concept of modular design is also used in the development of the proposed system. The wireless multi-channel EEG acquisition module can easily communicate with any type of commercial tablet via Bluetooth communication to increase acceptability for a large population of users. Therefore, the design of the BCI-based smart multimedia controller can be viewed as a completely innovative system prototype, and can be applied in different researches of music biofeedback in the future.
